# beta-1 Integrins mediate tumour cell adhesion to quiescent endothelial cells in vitro.

**DOI:** 10.1038/bjc.1996.627

**Published:** 1996-12

**Authors:** E. A. Price, D. R. Coombe, J. C. Murray

**Affiliations:** University of Nottingham Laboratory of Molecular Oncology, UK.

## Abstract

**Images:**


British Journal of Cancer (1996) 74, 1762-1766
© 1996 Stockton Press All rights reserved 0007-0920/96 $12.00

SHORT COMMUNICATION

### Beta-1 integrins mediate tumour cell adhesion to quiescent endothelial cells *in vitro*

EA Price[1], DR Coombe[2] and JC Murray[1]

[1]*University of Nottingham Laboratory of Molecular Oncology, CRC Department of Clinical Oncology, City Hospital, Nottingham, NG5 1PB, UK;* [2]*TVW Telethon Institute for Child Health Research, PO Box 855, West Perth, WA 6872, Australia.*

**Summary** Metastatic spread of some solid tumours is thought to depend upon the adhesion of tumour cells to the vascular endothelium followed by extravasation into surrounding tissues. We investigated the role of $\beta 1$ integrins in the adhesion of the breast adenocarcinoma cell line MDA-MB-231 and the melanoma cell line RPMI-7951 to quiescent human umbilical vein endothelial cells (HUVEC) *in vitro*. In the course of adhesion assays, tumour cells were observed to adhere to quiescent HUVEC monolayers, particularly at endothelial cell-cell junctions. Immunohistochemistry revealed concentration of $\beta 1$ integrin expression at these sites. Adhesion was reduced by pretreatment of either tumour cells or HUVEC with antibodies against $\beta 1$ integrins. Simultaneous treatment of HUVECs and tumour cells with these antibodies produced an additive blocking effect, consistent with a heterotypic adhesion mechanism. Our data suggest that tumour cell and endothelial $\beta 1$ integrins may play a crucial role in the arrest and migration of tumour cells through the vascular endothelium in the absence of endothelial 'activation'.

**Keywords:** endothelium; metastasis; $\beta 1$ integrin; adhesion

The interaction of tumour cells with endothelial cells lining the microvasculature is an important step in the metastatic cascade. A variety of endothelial and tumour adhesion molecules have been implicated in this process, and much attention has focused on the role of the integrin family of receptors (Honn and Tang, 1992). These transmembrane glycoproteins are composed of two non-covalently associated subunits termed $\alpha$ and $\beta$. There are at least 14 $\alpha$-subunits (150-200 kDa) and eight $\beta$-subunits (90-210 kDa). The $\alpha$-subunits can associate with more than one type of $\beta$-unit, leading to a wide range of integrin dimers (Hynes, 1992; Albelda and Buck, 1990). Ligand binding is dependent upon divalent cations (Gailit and Ruoslahti, 1988). The ligand recognised by integrins depends upon the particular $\alpha$- and $\beta$-subunits within the complex, and requires both subunits (Buck *et al.*, 1986). Endothelial integrins are involved in adhesion to extracellular matrix (ECM) molecules and in maintaining the integrity of the monolayer. Endothelial cells express $\beta 1$, $\beta 3$ and $\beta 5$ chains. The $\beta 1$ integrins, which are expressed on a wide range of cell types, dimerise with $\alpha$s 1-6 and thereby mediate cellular adhesion to laminin, collagen and fibronectin (Dejana, 1993). Integrins are also expressed on a variety of tumour cells, and changes in their expression may play a role in malignant progression and metastasis (Albelda, 1993; Juliano and Varner, 1993).

In addition to mediating adhesion to extracellular matrix components, beta-1 integrins have also been reported to play a role in tumour cell-endothelial cell adhesion. Lauri *et al.* (1991) found that $\alpha 5\beta 1$ expressed by tumour cell lines mediated their adhesion to interleukin 1 (IL-1)-activated human umbilical vein endothelial cells (HUVEC); treatment of different tumour cell lines with polyclonal antiserum against $\alpha 5\beta 1$ abrogated the enhanced adhesion. Some doubt surrounds the physiological significance of adhesion of tumour cells to endothelial cells activated by inflammatory cytokines, as this leads to acute up-regulation of a number of potential adhesion pathways. We have investigated the expression of $\beta 1$ integrins on two potentially metastatic tumour cell lines and non-activated endothelial cell monolayers, and their role in mediating cell-cell adhesion.

Correspondence: JC Murray
Received 6 February 1996; revised 20 June 1996; accepted 2 July 1996

### Materials and methods

### *Cells and cell culture*

The MDA-MB-231 human breast adenocarcinoma cell line and the RPMI-7951 human melanoma line (American Tissue Type Culture Collection, Rockville, MD, USA) were used in all experiments. HUVEC were isolated and cultured according to the method of Jaffe *et al.* (1973). Confluent cultures of HUVECs were used at passage 3.

### *Tumour cell/endothelial cell adhesion assay*

Tumour cell adhesion was measured as previously described (Price *et al.*, 1995). Briefly, HUVEC were grown on 96-well gelatin-coated plates until confluent. Subconfluent tumour cells were suspended with 10 mM EDTA (pH 7.0) and incubated in this solution for 1 h at 37°C. The released tumour cells were washed three times in Medium 199 supplemented with 0.1% bovine serum albumin (BSA) (assay medium) and then fluorescence labelled by incubation in 40 $\mu g\ ml^{-1}$ carboxy-fluorescein diacetate (CFDA; Sigma) in the same medium for 30 min at 37°C. After three washes in assay medium, cells were resuspended to give $1 \times 10^6$ cells $ml^{-1}$, and 50 $\mu l$ of cell suspension was added to each well of washed HUVEC monolayers. The plates were incubated for 30 min at 37°C to allow adhesion before washing the wells three times with phosphate-buffered saline (PBS). The cellular contents of each well were dissolved with sodium dodecylsulphate (50 $\mu l$ per well, 0.2%; Sigma, Poole, UK) to release the fluorescent marker. Lysates were collected and combined with two further washings with 50 $\mu l$ of PBS/A. Fluorescence of the cell lysates was determined with a Fluoroskan II plate reader (Labsystems, Basingstoke, UK) using a 485 nm excitation filter and a 538 nm emission filter. Relative adhesion was calculated as follows:

$$\text{Relative adhesion} = \frac{\text{Fluorescence of sample}}{\text{Fluorescence of untreated cells attached to untreated HUVEC}} \times 100$$

Data are expressed as the mean ± s.e.m. of at least three experiments performed in quadruplicate. Statistical analysis of the significance of observed differences between groups was carried out using Student's *t* test.

*Treatment with blocking antibodies*

A rat monoclonal antibody recognising $\beta 1$ integrins was obtained from Becton Dickinson (anti-CD29, clone 13). Mouse anti-human VCAM-1 (clone 1.G11B1) and mouse anti-human VLA-4 (CDw49d, clone HP2/1 which recognises the $\alpha 4$ integrin) were obtained from Serotec (Kidlington, Oxford, UK). Tumour cells were detached from the culture flasks with 10 mM EDTA, and washed twice before incubation with antibodies. Cells ($5 \times 10^6$) were incubated in 200 $\mu$l of antibody solution (10 $\mu$g ml$^{-1}$) for 30 min at 4°C. After antibody treatment, cells were washed, resuspended to a working dilution of $8 \times 10^6$ ml$^{-1}$ and used in adhesion assays. To treat HUVEC with antibody, wells containing monolayers were incubated with 50 $\mu$l of antibody (10 $\mu$g ml$^{-1}$) for 30 min at room temperature.

*Flow cytometry*

Before primary antibody binding, cells were incubated in 0.25 ml of 2% normal goat serum (NGS, Sigma) for 20 min at 4°C to block non-specific interactions with the secondary antibody. Suspended tumour cells ($5 \times 10^6$) were then incubated in 150 $\mu$l of primary antibody solution (10 $\mu$g ml$^{-1}$) for 45 min at 4°C. This was followed by incubation in 200 $\mu$l of appropriate FITC-conjugated secondary antibody (1 : 50) for 30 min at 4°C. After staining, cells were fixed in 1.5 ml of 4% paraformaldehyde (BDH, Derby, UK). Control samples were incubated with PBS/A in place of primary antibody. A Becton Dickinson FACScan was used to analyse antibody binding.

*Immunohistochemistry of HUVEC*

Staining was performed on fixed, non-permeabilised monolayers of HUVEC grown to confluence on glass tissue culture slides (Nunc, Naperville, IL, USA). Cells were washed with PBS/A and fixed in 0.05% glutaraldehyde for 30 min at 37°C. Monolayers were incubated with a 1 : 9 solution of normal goat serum in PBS/A for 25 min at room temperature to block non-specific binding of the secondary antibody. Monoclonal rat anti-CD29 ($\beta 1$ integrin) was used at 10 $\mu$g ml$^{-1}$ for 75 min at 4°C. The secondary antibody was a FITC-conjugated goat anti-rat monoclonal (Sigma), used at a 1 : 50 dilution. Cells were incubated with secondary antibody for 30 min at 4°C. Slides were mounted with a 1 : 1 solution of glycerol (Sigma) and PBS/A, and viewed with an Optiphot microscope equipped with epifluorescence (Nikon, Melville, NY, USA).

*Silver staining of HUVEC*

HUVECs were grown to confluence in 24-well dishes precoated with rat tail tendon collagen, which was allowed to gel (Boehringer Mannheim, Germany). Gels were formed according to the manufacturer's instructions. Adhesion assays

**Figure 1** Fluorescence histograms demonstrating the expression of $\beta 1$ integrins (MAb 13) and VLA-4 (CDw49d, MAb HP2/1) by MDA-MB-231 and RPMI-7951 tumour cells obtained by flow cytometry. Control profiles (open curves) were obtained by omitting primary antibodies. (**a**) MDA-MD-231 + anti-$\beta 1$ integrin (**b**) RPMI-7951 + anti-$\beta 1$ integrin. (**c**) MDA-MB-231 + anti-VLA-4. (**d**) RPMI-7951 + anti-VLA-4.

were carried out using tumour cells disaggregated with 10 mM EDTA. After allowing the tumour cells to adhere to HUVEC monolayers for 30 min, the wells were washed three times, fixed with 0.1% glutaraldehyde in PBS/A and the cultures stained with silver nitrate as described by Furie *et al.* (1984).

### Results

FACScan analysis demonstrated high levels of cell surface expression of $\beta 1$ integrins on both tumour cell lines (Figure 1). However, only the RPMI-7951 line expressed the $\alpha 4\beta 1$ integrin VLA-4, which is the ligand for VCAM-1 on activated endothelial cells (Rice and Bevilacqua, 1989). Immunofluorescence staining of intact HUVEC monolayers confirmed the expression of $\beta 1$ integrins by endothelial cells; staining was particularly intense at endothelial cell–cell junctions (Figure 2).

We previously demonstrated that both MDA-MB-231 and RPMI-7951 tumour cells adhere to monolayers of quiescent HUVEC in preference to gelatin, in the absence of cytokine activation (Price *et al.*, 1995). Under these conditions, differential adhesion to HUVEC was maximal after 30 min, when 40%±3% ($n=12$) of MDA-MB-231 and 47%±2% ($n=12$) of RPMI-7951 tumour cells are adherent to HUVEC monolayers. Consequently, all measurements were made after 30 min of incubation and adhesion data are hereafter expressed as per cent control adhesion at 30 min.

We observed that blocking of cell-surface $\beta 1$ integrins, by preincubation of either HUVECs or tumour cells with anti-CD29 (MAb 13), caused significant reductions in adhesion (Figure 3). When both HUVECs and tumour cells were pretreated simultaneously with antibody, the overall reduction in adhesion obtained was approximately equal to the sum of the separate changes. Although RPMI-7951 cells express the $\beta 1$ integrin VLA-4 (see Figure 1), blocking tumour cells with MAb HP2/1 or HUVEC with MAb 1.G11B1 against VCAM-1 (the endothelial receptor for VLA-4) did not reduce the adhesion of RPMI-7951 cells to HUVEC (data not shown). This suggests that the adhesion of the RPMI-7951 cell line to the quiescent HUVEC is not mediated by tumour cell-surface VLA-4 interacting with VCAM-1 on HUVECs, and that alternative $\beta 1$ integrins are involved. MDA-MB-231 cells do not express VLA-4.

During the course of the adhesion experiments we observed that, when incubated with HUVEC monolayers, tumour cells tended to accumulate and adhere preferentially at endothelial cell–cell junctions. To confirm this observation, we made a silver-stained preparation of MDA-MB-231 cells incubated with HUVEC for 30 min. This staining technique highlights glycosaminoglycans present at the cell–cell junctions, giving a 'paving stone' effect. Tumour cells can be clearly seen lining up along cell junctions and are rarely observed adhering to cell surfaces remote from such junctions (Figure 4).

**Figure 2** Immunofluorescent staining of confluent HUVEC with anti-$\beta 1$ integrin MAb 13. (**a**) Positive staining showing enrichment of $\beta 1$ integrins at endothelial cell–cell junctions. (**b**) Control with primary antibody omitted (magnification ×400).

**Figure 3** The effects of pretreating MDA-MB-231 or RPMI-7951 tumour cells with anti-$\beta 1$ integrin (MAb 13) on adhesion to quiescent monolayers of HUVECs; ☐, adhesion of untreated tumour cells to untreated HUVECs. ☐, HUVECs treated with antibody; ▨, tumour cells treated with antibody; ▩, HUVEC and tumour cells treated with antibody. $*P<0.05$, $n=12$.

**Figure 4** Localisation of tumour cells to endothelial cell–cell junctions in a monolayer of human umbilical vein endothelial cells. MDA-MB-231 cells were allowed to attach as described in Materials and methods. Endothelial cell–cell junctions are silver stained; cells are counterstained with Wright–Giemsa. Arrows indicate examples of tumour cells clearly associated with endothelial cell–cell junctions (magnification ×200).

### Discussion

Beta-1 integrins are expressed at high levels on the surface of both the MDA-MB-231 and the RPMI-7951 cell lines. In addition, we found that these integrins are expressed by HUVEC, on which they tend to be concentrated at cell–cell junctions, as shown by Lampugnani *et al.* (1991). We also observed that the tumour cells tend to localise at such sites during adhesion assays. Beta-1 integrins on both cell types appear to function as adhesion molecules in our model, as blocking these integrins on either the tumour cells or HUVECs led to significant reductions in adhesion. This adhesion is not based upon a homotypic interaction, as simultaneous blocking of integrins on both tumour cell lines and HUVECs produced an additive effect, which suggests a heterotypic interaction between these cells.

Beta-1 integrin expression on endothelial cells has previously been demonstrated (Lampugnani *et al.*, 1991). They are also expressed on normal breast epithelium, on their neoplastic counterparts at reduced levels (Mechtersheimer *et al.*, 1993), and on melanoma cells (Elices and Hemmler, 1989). Recently Maemura *et al.* (1995), while studying $\alpha2\beta1$ expression using a range of normal and malignantly transformed breast epithelial cell lines, showed that this integrin is universally expressed but that its function as a receptor for laminin is altered in a manner which appears to be correlated with metastatic potential. Therefore, while highly metastatic breast cell lines such as the MDA-MB-231 line (used in this study) express this integrin, its affinity for laminin is reduced. This suggests that not tumour cell $\alpha2\beta1$ but rather some other $\beta1$ integrin may be involved in adhesion to endothelial cells in our model. Alternatively, a ligand other than laminin on the endothelium is involved.

Many integrins are capable of interacting with multiple ligands (Hynes, 1992); indeed, the ligand recognised by an integrin may depend upon which cell type it is expressed on (Elices and Hemler, 1989). Nor is there any reason to assume that while $\beta1$ integrins on both tumour and endothelial cells are involved in adhesion they interact with the same ligands on either cell. As stated earlier, $\beta1$ integrins mediate the adhesion of cells to extracellular matrix proteins; the integrin $\alpha5\beta1$, for example, mediates adhesion to fibronectin. However, MDA-MB-231 cells shed much of their newly synthesised fibronectin into the culture medium (Incardona *et al.*, 1993), discounting fibronectin as the tumour ligand for endothelial integrins. Lauri *et al.* (1991) found that endothelial fibronectin was not involved in the $\beta1$ integrin-mediated adhesion of various tumour cell lines to HUVEC. Other extracellular matrix molecules such as epiligrin (Carter *et al.*, 1991) and collagen (Wayner and Carter, 1987) cannot be ruled out as potential ligands in this model. Laminin may also be a ligand (D'Ardenne *et al.*, 1991), although possibly for $\beta1$ integrins other than $\alpha2\beta1$, as discussed above.

Bliss *et al.* (1995) recently reported the inhibition of adhesion of two breast cancer cell lines to endothelial cells by antibodies against the $\alpha6\beta1$ integrin (a laminin receptor) in one case and $\alpha5\beta1$ (a fibronectin receptor) in another. These authors concluded that tumour cells interact with 'luminal components of the extracellular matrix' and not directly with the endothelial cell surface. These findings offer a potential explanation for our observations on the localisation of tumour cells to endothelial cell–cell junctions.

Another potential ligand for the $\beta1$ integrins, expressed on both tumour and endothelial cells, is thrombospondin (TSP) (Bornstein, 1995). MDA-MB-231 cells synthesise TSP and express high-affinity TSP receptors (Incardona *et al.*, 1993); our preliminary data show that the RPMI-7951 cell line also expresses TSP (data not shown). Additionally, HUVEC express both TSP receptors and TSP clusters on the apical surface, which mediate the adhesion of MCF-7 breast adenocarcinoma cells (Incardona *et al.*, 1995).

We may surmise that the unidentified endothelial ligand, like the $\beta1$ integrins, must be expressed predominantly at intercellular junctions. The transmembrane glycoprotein CD31 (PECAM-1), which is highly enriched in endothelial junctions, may play a role. CD31 participates in heterophilic interactions with proteoglycans (Muller *et al.*, 1992), and it has been postulated to play a role in tumour cell adhesion and migration through blood vessel walls (Honn and Tang, 1992; Tang *et al.*, 1993). CD31 does not interact in a ligand/counterligand manner with $\beta1$ integrins; however CD31 expressed on T-cell subsets has been shown to act as a preferential amplifier of $\beta1$ integrin-mediated adhesion to endothelial cells (Tanaka *et al.*, 1992). This suggests that the presence of CD31 at the endothelial cell–cell junction would tend to favour integrin-based interactions, preferentially amplifying the activity of $\beta1$ integrins at this site.

We conclude that $\beta1$ integrins expressed at endothelial cell–cell junctions and on the surface of the MDA-MB-231 and RPMI-7951 tumour cell lines mediate heterotypic adhesion. The ligands remain obscure. Putting these observations into a physiological context, the model suggests that such interactions localise tumour cells to sites where they may more readily initiate the process of extravasation from the vasculature through the blood vessel wall and into the extravascular space. Furthermore, such a mechanism is more physiologically relevant in that it is dependent upon constitutively expressed molecules and not upon prior 'activation' of endothelial cells.

**Acknowledgements**
This work was supported by a Cancer Research Campaign Studentship to EAP and an equipment grant from the Dr Hadwen Trust for Humane Research. Special thanks are also due to the members of the St Albans CRC Fund-Raising Committee.

### References

ALBELDA SM. (1993). Role of integrins and other cell adhesion molecules in tumour progression and metastasis. *Lab. Invest.*, **68**, 4–17.

ALBELDA SM AND BUCK CA. (1990). Integrins and other cell adhesion molecules. *FASEB J.*, **4**, 2868–2880.

BLISS RD, KIRBY JA, BROWELL DA AND LENNARD TWJ. (1995). The role of $\beta1$ integrins in adhesion of two breast carcinoma cell lines to a model endothelium. *Clin. Exp. Metastasis*, **13**, 173–183.

BORNSTEIN P. (1995). Diversity is inherent in matricellular proteins: an appraisal of thrombospondin 1. *J. Cell Biol.*, **130**, 503–506.

BUCK CA, SHE E, DUGGAN K AND HORWITZ AF. (1986). Integrin (The CSAT antigen): functionality requires oligomeric integrity. *J. Cell Biol.*, **103**, 2421–2428.

CARTER WG, RYAN MC AND GAHR PJ. (1991). Epiligrin, a new cell adhesion ligand for integrin $\alpha3\beta1$ in epithelial basement membranes. *Cell*, **65**, 599–610.

D'ARDENNE AJ, RICHMAN PI, HORTON MA, MCAULAY AE AND JORDAN S. (1991). Coordinate expression of the alpha-6 integrin laminin receptor sub-unit and laminin in breast cancer. *J. Pathol.*, **165**, 213–220.

DEJANA E. (1993). Endothelial cell adhesive receptors. *J. Cardiovasc. Pharmacol.*, **21**, S18–S21.

ELICES MJ AND HEMLER ME. (1989). The human integrin VLA-2 is a collagen receptor on some cells and a collagen/laminin receptor on others. *Proc. Natl Acad. Sci. USA*, **86**, 9906–9910.

FURIE MB, CRAMER EB, NAPRSTEK BL AND SILVERSTEIN SC. (1984). Cultured endothelial cell monolayers that restrict the passage of macromolecules and electrical current. *J. Cell Biol.*, **98**, 1033–1041.

GAILIT J AND RUOSLAHTI E. (1988). Regulation of the fibronectin receptor affinity by divalent cations. *J. Biol. Chem.*, **263**, 12927–12932.

HONN KV AND TANG DG. (1992). Adhesion molecules and tumour cell interactions with endothelium and subendothelial matrix. *Cancer Metastasis Rev.*, **11**, 353–375.

HYNES RO. (1992). Integrins: versatility, modulation, and signaling in cell adhesion. *Cell*, **69**, 11–25.

INCARDONA F, CALVO F, FUAVEL-LAFEVE F, LEGRAND Y AND LEGRAND C. (1993). Involvement of thrombospondin in the adherance of human breast adenocarcinoma cells: a possible role in the metastatic process. *Int. J. Cancer*, **55**, 471–477.

INCARDONA FL, LEWALLE JM, MORANDI V, LAMBERT S, LEGRAND Y, FOIDART JM AND LEGRAND C. (1995). Thrombospondin modulates human breast adenocarcinoma cell adhesion to human vascular endothelial cells. *Cancer Res.*, **55**, 166–173.

JAFFE EA, NACHMAN RL, BECKER CG AND MINICK CR. (1973). Culture of human endothelial cells derived from umbilical veins. Identification by morphologic and immunologic criteria. *J. Clin. Invest.*, **52**, 2745–2756.

JULIANO RL AND VARNER JA. (1993). Adhesion molecules in cancer: the role of integrins. *Curr. Opin. Cell Biol.*, **5**, 812–818.

LAMPUGNANI MG, RESNATI M, DEJANA E AND MARCHISIO PC. (1991). The role of integrins in the maintenance of endothelial monolayer integrity. *J. Cell Biol.*, **112**, 479–490.

LAURI D, MARTIN-PADURA I, BIONDELLI T, ROSSI G, BERNASCONI S, GIAVAZZI R, PASSERINI F, VAN HINSBERGH V AND DEJANA E. (1991). Role of $\beta 1$ integrins in tumour cell adhesion to cultured human endothelial cells. *Lab. Invest.*, **65**, 525–531.

MAEMURA M, AKIYAMA SK, WOODS VL AND DICKSON RB. (1995). Expression and ligand binding of $\alpha 2\beta 1$ integrin on breast carcinoma cells. *Clin. Exp. Metastasis*, **13**, 223–235.

MECHTERSHEIMER G, MUNK M, BARTH K, KORETZ K AND MOLLER P. (1993). Expression of $\beta 1$ integrins in non-neoplastic mammary epithelium, fibroadenoma and carcinoma of the breast. *Virchows Archiv. A*, **422**, 203–210.

MULLER WA, BERMAN ME, NEWMAN PJ, DELISSER HM AND ALBELDA SM. (1992). A heterophilic adhesion mechanism for platelet/endothelial cell adhesion molecules. *J. Exp. Med.*, **175**, 1401–1404.

PRICE EA, COOMBE DR AND MURRAY JC. (1995). A simple fluorometric assay for quantifying the adhesion of tumour cells to endothelial monolayers. *Clin. Exp. Metastasis*, **13**, 155–164.

RICE GE AND BEVILACQUA MP. (1989). An inducible endothelial cell surface glycoprotein mediates melanoma adhesion. *Science*, **246**, 1303–1306.

TANAKA Y, ALBELDA SM, HORGAN KJ, VAN SEVENTER GA, SHIMIZU Y, NEWMAN W, HALLAM J, NEWMAN PJ, BUCK CA AND SHAW S. (1992). CD31 expressed on distinctive T cell subsets is a preferential amplifier of $\beta 1$ integrin-mediated adhesion. *J. Exp. Med.*, **176**, 245–253.

TANG DG, CHEN YQ, NEWMAN PJ, SHI L, GAO X, DIGLIO CA AND HONN KV. (1993). Identification of PECAM-1 in solid tumor cells and its potential involvement in tumor cell adhesion to endothelium. *J. Biol. Chem.*, **268**, 22883–22894.

WAYNER EA AND CARTER WG. (1987). Identification of multiple adhesion receptors for collagen and fibronectin in human fibrosarcoma cells possessing unique $\alpha$ and common $\beta$ subunit. *J. Cell Biol.*, **105**, 1873–1874.


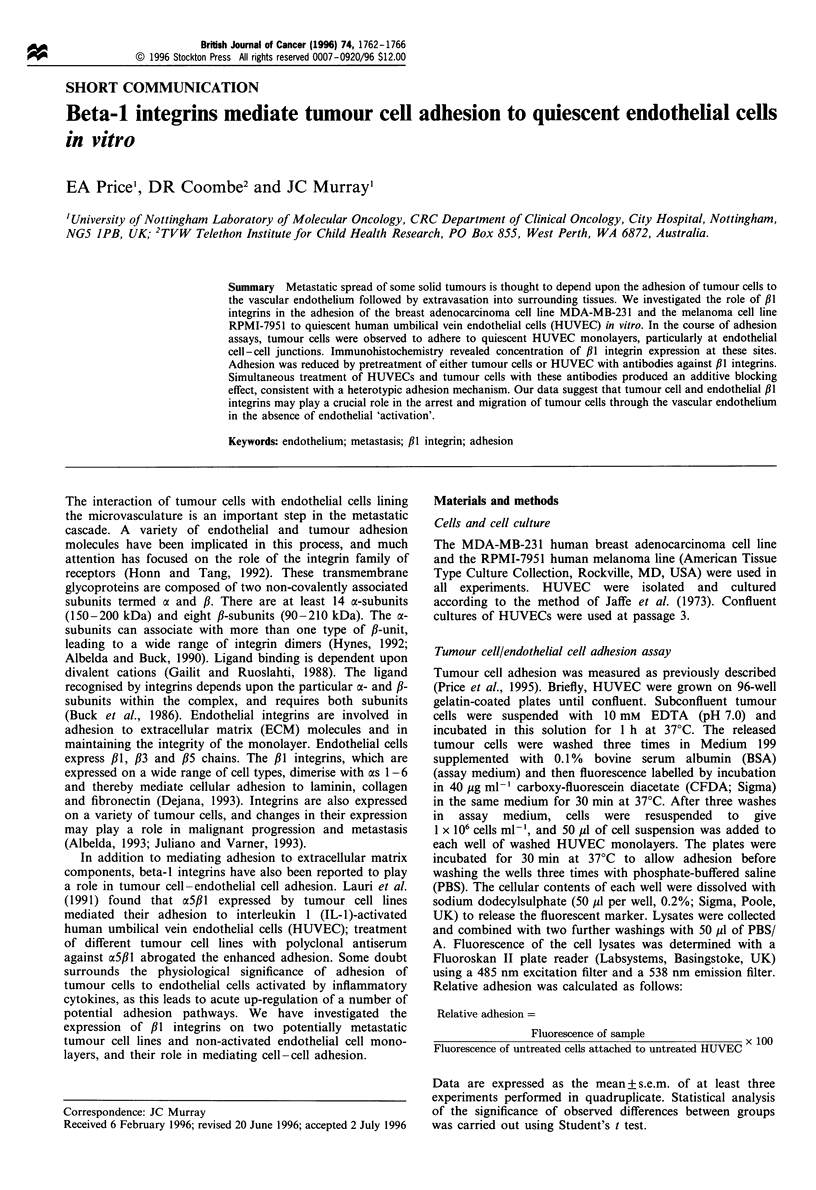

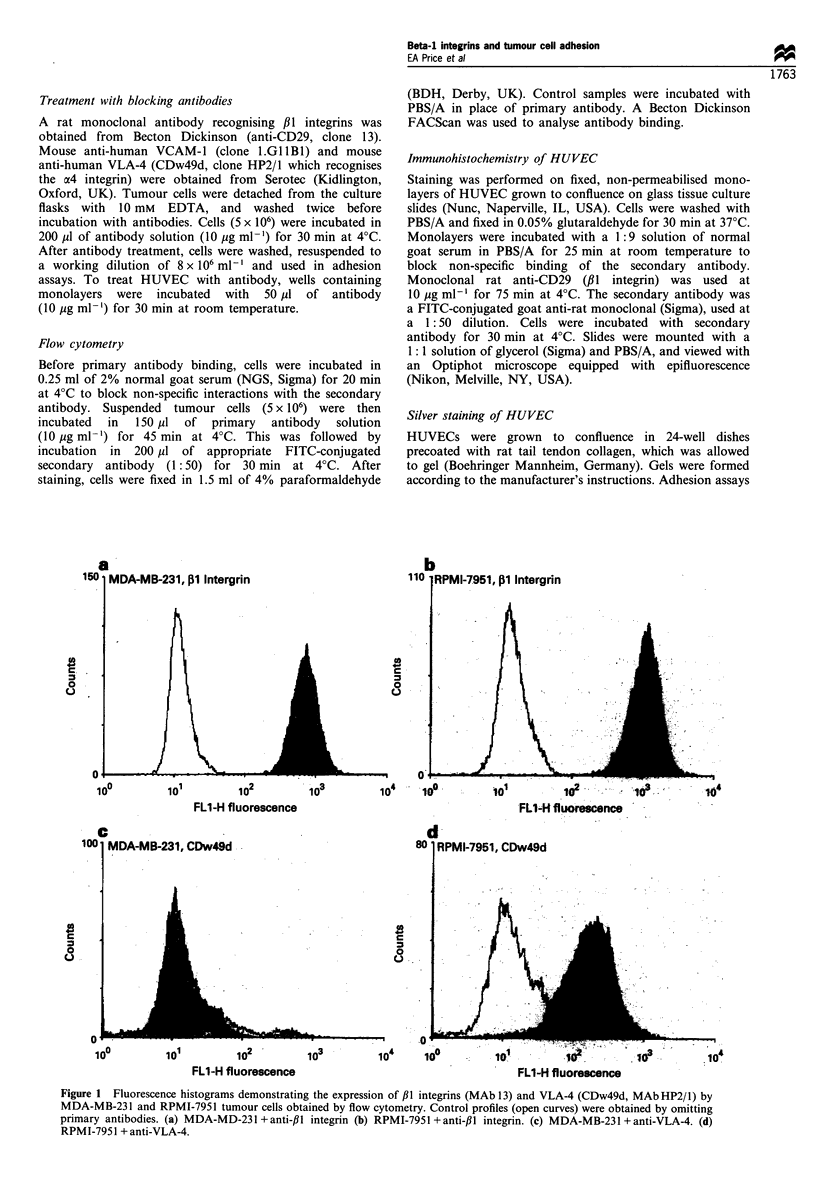

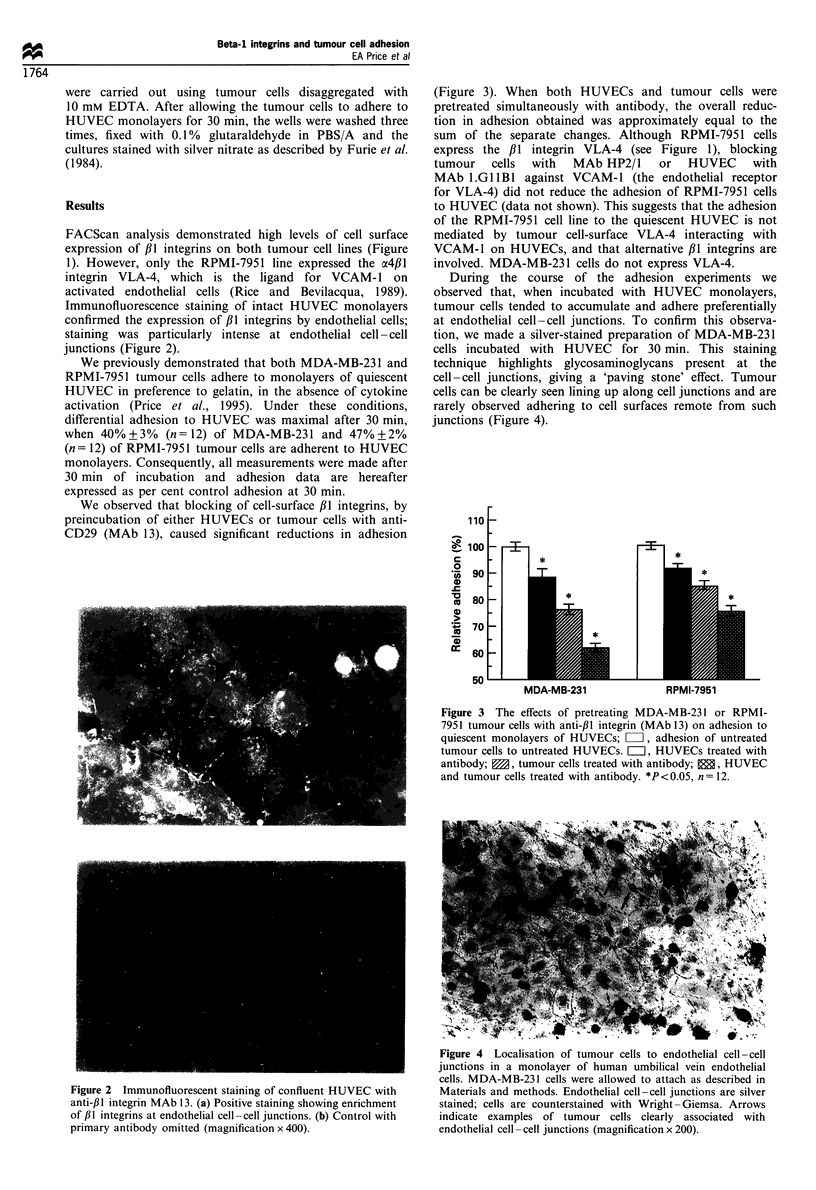

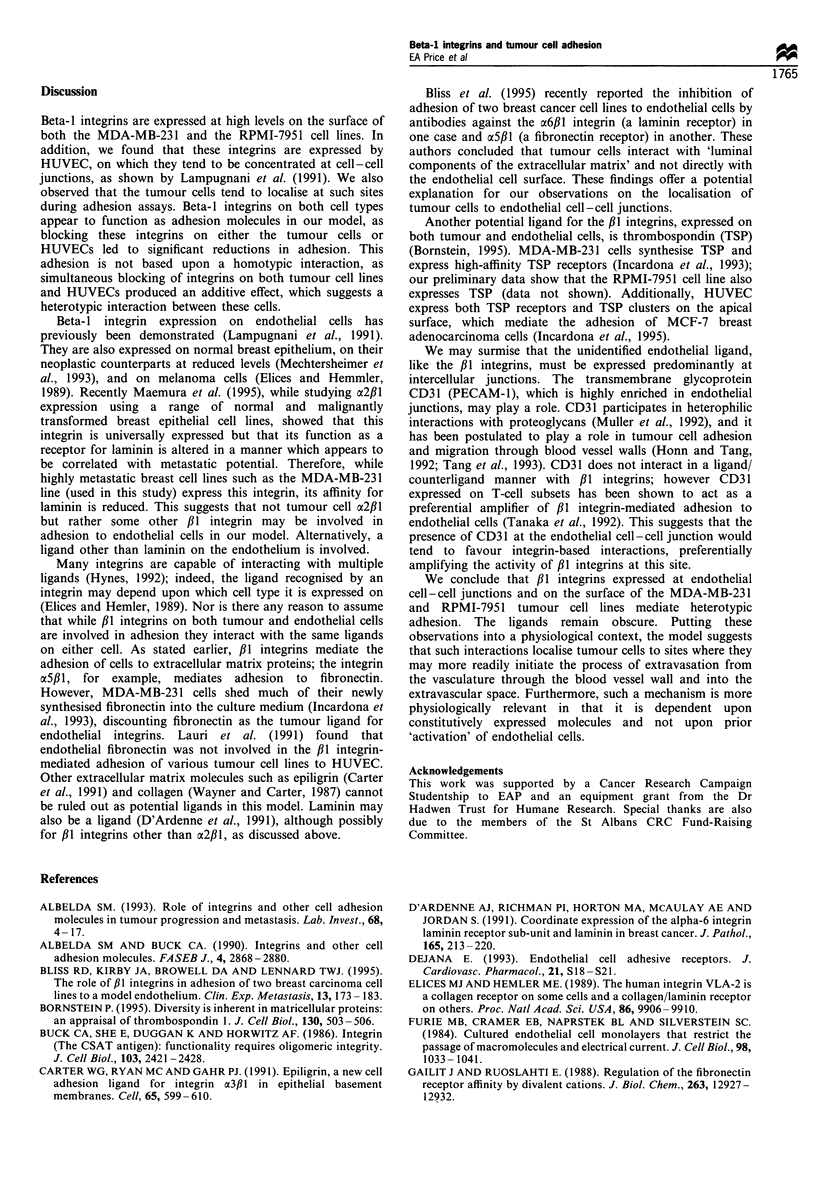

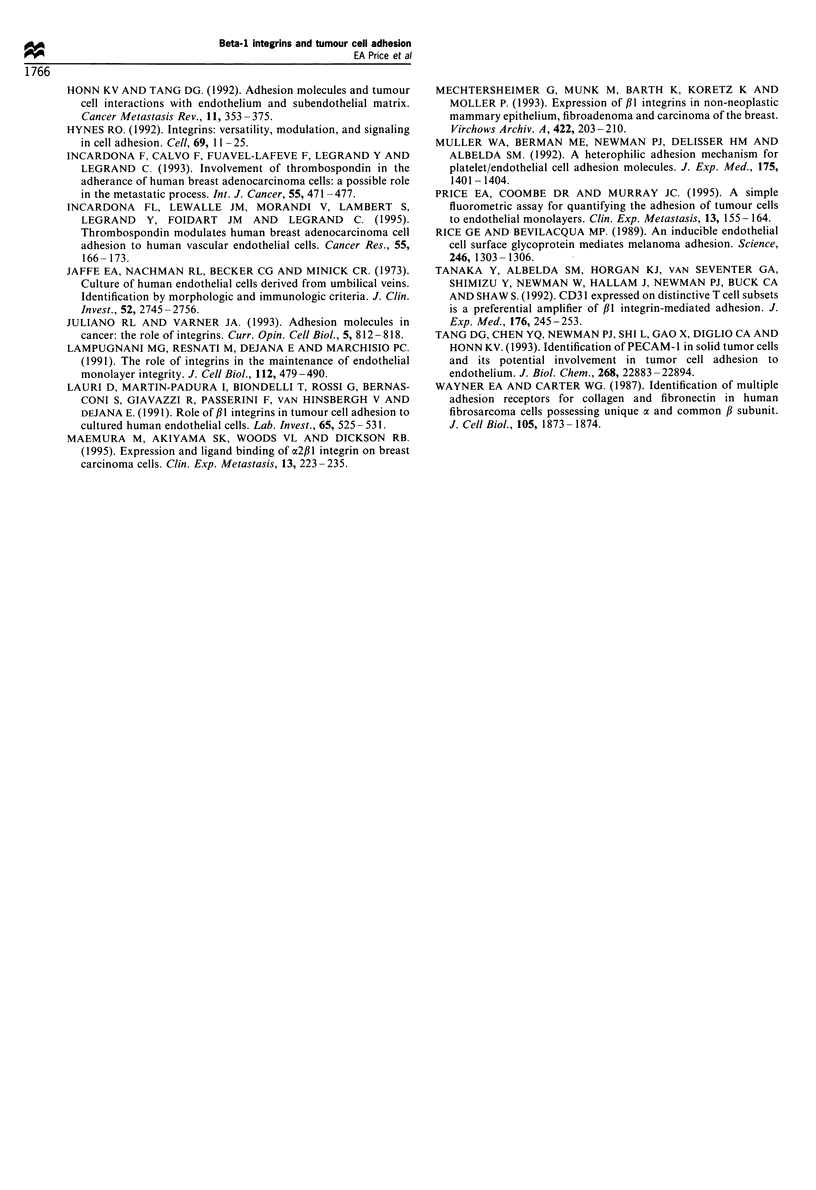


## References

[OCR_00495] Albelda S. M., Buck C. A. (1990). Integrins and other cell adhesion molecules.. FASEB J.

[OCR_00490] Albelda S. M. (1993). Role of integrins and other cell adhesion molecules in tumor progression and metastasis.. Lab Invest.

[OCR_00499] Bliss R. D., Kirby J. A., Browell D. A., Lennard T. W. (1995). The role of beta 1 integrins in adhesion of two breast carcinoma cell lines to a model endothelium.. Clin Exp Metastasis.

[OCR_00501] Bornstein P. (1995). Diversity of function is inherent in matricellular proteins: an appraisal of thrombospondin 1.. J Cell Biol.

[OCR_00505] Buck C. A., Shea E., Duggan K., Horwitz A. F. (1986). Integrin (the CSAT antigen): functionality requires oligomeric integrity.. J Cell Biol.

[OCR_00512] Carter W. G., Ryan M. C., Gahr P. J. (1991). Epiligrin, a new cell adhesion ligand for integrin alpha 3 beta 1 in epithelial basement membranes.. Cell.

[OCR_00518] D'Ardenne A. J., Richman P. I., Horton M. A., Mcaulay A. E., Jordan S. (1991). Co-ordinate expression of the alpha-6 integrin laminin receptor sub-unit and laminin in breast cancer.. J Pathol.

[OCR_00523] Dejana E. (1993). Endothelial cell adhesive receptors.. J Cardiovasc Pharmacol.

[OCR_00525] Elices M. J., Hemler M. E. (1989). The human integrin VLA-2 is a collagen receptor on some cells and a collagen/laminin receptor on others.. Proc Natl Acad Sci U S A.

[OCR_00530] Furie M. B., Cramer E. B., Naprstek B. L., Silverstein S. C. (1984). Cultured endothelial cell monolayers that restrict the transendothelial passage of macromolecules and electrical current.. J Cell Biol.

[OCR_00538] Gailit J., Ruoslahti E. (1988). Regulation of the fibronectin receptor affinity by divalent cations.. J Biol Chem.

[OCR_00548] Honn K. V., Tang D. G. (1992). Adhesion molecules and tumor cell interaction with endothelium and subendothelial matrix.. Cancer Metastasis Rev.

[OCR_00553] Hynes R. O. (1992). Integrins: versatility, modulation, and signaling in cell adhesion.. Cell.

[OCR_00558] Incardona F., Calvo F., Fauvel-Lafeve F., Legrand Y., Legrand C. (1993). Involvement of thrombospondin in the adherence of human breast-adenocarcinoma cells: a possible role in the metastatic process.. Int J Cancer.

[OCR_00564] Incardona F., Lewalle J. M., Morandi V., Lambert S., Legrand Y., Foidart J. M., Legrand C. (1995). Thrombospondin modulates human breast adenocarcinoma cell adhesion to human vascular endothelial cells.. Cancer Res.

[OCR_00570] Jaffe E. A., Nachman R. L., Becker C. G., Minick C. R. (1973). Culture of human endothelial cells derived from umbilical veins. Identification by morphologic and immunologic criteria.. J Clin Invest.

[OCR_00576] Juliano R. L., Varner J. A. (1993). Adhesion molecules in cancer: the role of integrins.. Curr Opin Cell Biol.

[OCR_00578] Lampugnani M. G., Resnati M., Dejana E., Marchisio P. C. (1991). The role of integrins in the maintenance of endothelial monolayer integrity.. J Cell Biol.

[OCR_00586] Lauri D., Martin-Padura I., Biondelli T., Rossi G., Bernasconi S., Giavazzi R., Passerini F., Van Hinsbergh V., Dejana E. (1991). Role of beta 1 integrins in tumor cell adhesion to cultured human endothelial cells.. Lab Invest.

[OCR_00591] Maemura M., Akiyama S. K., Woods V. L., Dickson R. B. (1995). Expression and ligand binding of alpha 2 beta 1 integrin on breast carcinoma cells.. Clin Exp Metastasis.

[OCR_00597] Mechtersheimer G., Munk M., Barth T., Koretz K., Möller P. (1993). Expression of beta 1 integrins in non-neoplastic mammary epithelium, fibroadenoma and carcinoma of the breast.. Virchows Arch A Pathol Anat Histopathol.

[OCR_00600] Muller W. A., Berman M. E., Newman P. J., DeLisser H. M., Albelda S. M. (1992). A heterophilic adhesion mechanism for platelet/endothelial cell adhesion molecule 1 (CD31).. J Exp Med.

[OCR_00608] Price E. A., Coombe D. R., Murray J. C. (1995). A simple fluorometric assay for quantifying the adhesion of tumour cells to endothelial monolayers.. Clin Exp Metastasis.

[OCR_00613] Rice G. E., Bevilacqua M. P. (1989). An inducible endothelial cell surface glycoprotein mediates melanoma adhesion.. Science.

[OCR_00619] Tanaka Y., Albelda S. M., Horgan K. J., van Seventer G. A., Shimizu Y., Newman W., Hallam J., Newman P. J., Buck C. A., Shaw S. (1992). CD31 expressed on distinctive T cell subsets is a preferential amplifier of beta 1 integrin-mediated adhesion.. J Exp Med.

[OCR_00623] Tang D. G., Chen Y. Q., Newman P. J., Shi L., Gao X., Diglio C. A., Honn K. V. (1993). Identification of PECAM-1 in solid tumor cells and its potential involvement in tumor cell adhesion to endothelium.. J Biol Chem.

[OCR_00629] Wayner E. A., Carter W. G. (1987). Identification of multiple cell adhesion receptors for collagen and fibronectin in human fibrosarcoma cells possessing unique alpha and common beta subunits.. J Cell Biol.

